# Validating the 2023 FIGO staging system: A nomogram for endometrioid endometrial cancer and adenocarcinoma

**DOI:** 10.1002/cam4.7216

**Published:** 2024-05-16

**Authors:** Yifan Feng, Fulu Miao, Yuyang Li, Min Li, Yunxia Cao

**Affiliations:** ^1^ Department of Gynecology Oncology The First Affiliated Hospital of Anhui Medical University Hefei China; ^2^ NHC Key Laboratory of Study on Abnormal Gametes and Reproductive Tract (Anhui Medical University) Hefei Anhui China; ^3^ Key Laboratory of Population Health Across Life Cycle (Anhui Medical University), Ministry of Education of the People's Republic of China Hefei Anhui China

**Keywords:** endometrial cancer, FIGO, grade, nomogram, overall survival

## Abstract

**Background:**

To find the factors impacting overall survival (OS) prognosis in patients with endometrioid endometrial carcinoma (EEC) and adenocarcinoma and to establish a nomogram model to validate the 2023 International Federation of Obstetrics and Gynecology (FIGO) staging system for endometrial cancer.

**Methods:**

Data were obtained from the Surveillance, Epidemiology, and End Results (SEER) training cohort. An independent validation cohort was obtained from the First Affiliated Hospital of Anhui Medical University between 2008 and 2023. Cox regression analysis identified independent prognostic factors for OS in EEC and adenocarcinoma patients. A nomogram predicting OS was developed and validated utilizing the C‐index, calibration curves, receiver operating characteristic (ROC) curves, and decision curve analysis (DCA). The relationship between the tumor grade and prognosis of EEC and adenocarcinoma was quantified using net reclassification improvement (NRI), propensity score matching (PSM), and Kaplan–Meier curves.

**Results:**

Cox regression analysis identified age, race, marital status, tumor grade, tumor stage, tumor size, and chemotherapy as independent prognostic factors for OS. A nomogram for predicting OS was developed based on these factors. The C‐indexes for the OS nomogram was 0.743 and 0.720 for the SEER training set and external validation set, respectively. The area under the ROC (AUC) for the OS nomogram was 0.755, 0.757, and 0.741 for the SEER data subsets and 0.844, 0.719, and 0.743 for the external validation subsets. Calibration plots showed high concordance between the nomogram‐predicted and observed OS. DCA also demonstrated the clinical utility of the OS nomogram. NRI, PSM, and survival analyses revealed that tumor grade was the most important histopathological factor for EEC and adenocarcinoma prognosis.

**Conclusion:**

Seven independent prognostic variables for the OS of patients with EEC and adenocarcinoma were identified. The established OS nomogram has good predictive ability and clinical utility and validates the 2023 endometrial cancer FIGO staging system.

## 1 BACKGROUND

Endometrial cancer ranks as the sixth most common cancer in women, with an alarming 417,000 new cases reported globally in 2020.^1^ Despite these numbers, there is no entrenched screening program for endometrial cancer in the general female population or among particular high‐risk groups. The invasive nature of endometrial sampling makes wide‐scale screening unfeasible. Consequently, over the past three decades, a 132% spike in the prevalence of endometrial cancer has been observed.^2^ The increasing incidence of endometrial cancer is not limited to affluent populations, but disproportionately affects women of lower socioeconomic status globally, resulting in a considerable public health burden.^3^

The surgical stage, histological type, grade, and extent of organ involvement form the foundation for determining the stage and assessing the prognosis of endometrial cancer.^4^ The universally accepted staging guidelines, based on the TNM system of the Union for International Cancer Control (UICC) standards, are agreed upon by the International Federation of Obstetrics and Gynecology (FIGO).^4^, ^5^ The FIGO staging system is the method of choice for the clinical evaluation of patient prognosis with endometrial cancer.^6^

In 2023, the FIGO staging system for endometrial cancer underwent significant modifications from the 2009 system, centralizing histopathological findings in the reformed FIGO staging system.^7^ This revised stage definition incorporates pathologic variables such as histological classification, grade, and lymphovascular space invasion (LVSI), enabling more precise staging. Nevertheless, FIGO staging has clear limitations, including low accuracy, the exclusion of variables such as age and race, and poor performance in predicting individual survival risk.^8^, ^9^ A burgeoning trend involves the use of nomograms as predictive cancer models, owing to their ability to streamline a multitude of complex factors into an easily interpreted numerical prediction model that gauges the likelihood of certain outcomes.^10^

This study investigated the determinants influencing the prognosis of endometrioid endometrial cancer (EEC) and adenocarcinoma patients. We established a prognostic nomogram model and verified part of the 2023 FIGO staging system for endometrial cancer.

## 2 MATERIALS AND METHODS

### 2.1 Data source

This retrospective observational study utilized data from the Surveillance, Epidemiology, and End Results (SEER) program. Offering federally‐funded, publicly accessible cancer reports, the SEER database has no personal identification information linked to cases extracted for research purposes.^11^

To validate the model under study, an external validation set was constructed utilizing data acquired from the First Affiliated Hospital of Anhui Medical University from 2008 to 2023. All individuals included in this dataset were diagnosed by pathology with EEC and adenocarcinoma. We regularly followed up all these patients—evaluations were carried out every 3 months within the first 2 years, semiannually for the next 3 years, and annually thereafter. This study was approved by the First Affiliated Hospital of Anhui Medical University.

### 2.2 Inclusion criteria

**FIGURE 1:**
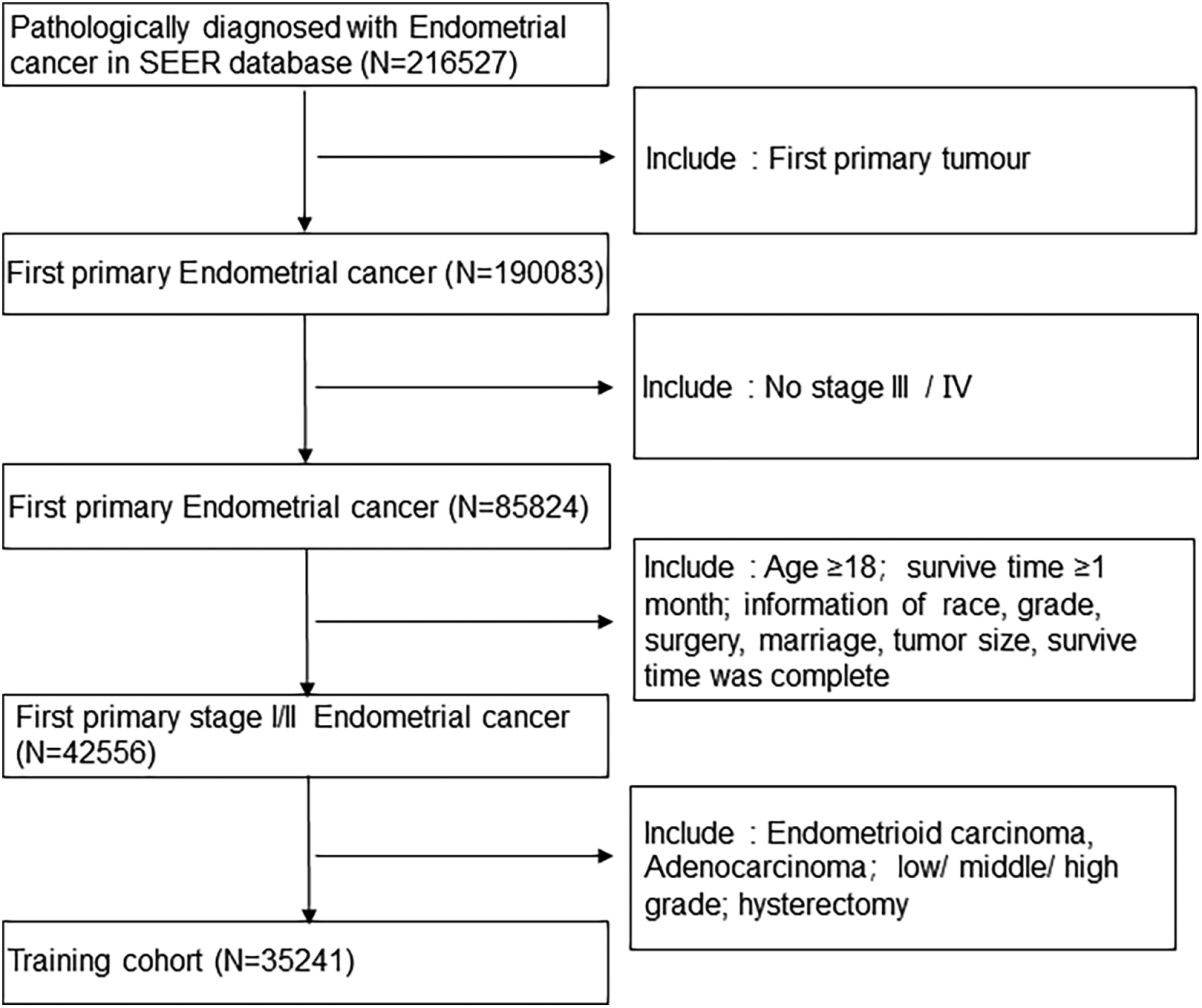
Study selection process.

The patient selection process is delineated in Figure 1. The inclusion criteria were patients who were diagnosed with EEC and adenocarcinoma between 2004 and 2015; who were registered within the SEER database; who had endometrial cancer as the primary tumor; who lacked stage III/IV disease; who were ≥18 years old; who had a survival duration greater than 1 month; and who had comprehensive information regarding race, tumor grade, marital status, tumor size, and survival month. The tumor grades were low, middle, and high. All patients were subjected to the fundamental total hysterectomy procedure.^12^, ^13^ The same stipulations were enforced for the external validation set.

### 2.3 Statistical analysis

Categorical variables are summarized using counts and percentages. Univariate and multivariate Cox regression analyses were performed to evaluate potential prognostic factors for EEC and adenocarcinoma, and hazard ratios (HRs) and 95% confidence intervals (CIs) were calculated. Overall survival (OS), defined as the interval from diagnosis to death or last follow‐up, was the primary endpoint. Significant prognostic factors identified by the Cox proportional hazards model were incorporated into a nomogram for predicting OS in EEC and adenocarcinoma patients. Nomogram performance was evaluated using Harrell's C‐index and receiver operating characteristic (ROC) curves. Calibration plots depicted the calibration between nomogram‐predicted and actual survival probabilities. Decision curve analysis (DCA) was run to determine the clinical utility of the nomogram by quantifying the net benefit across a range of threshold probabilities.

The effect of tumor grade on the prognosis of patients with EEC and adenocarcinoma was validated. The net reclassification improvement (NRI) was utilized to appraise enhancements in risk prediction and to evaluate the utility of the new model.^14^ Propensity score matching (PSM) was done via the caliper match method, utilizing logistic regression to match an array of variables (age, race, marital status, tumor size, radiotherapy, and chemotherapy) between the stage I high‐grade and stage II low/middle‐grade group. The chi‐squared test was run to scrutinize the heterogeneity of intergroup variables after PSM. Kaplan–Meier curves were drawn and the log‐rank tests run to compare OS between patients with stage I high‐grade disease and patients with stage II low/middle‐grade disease. All the statistical analyses and figure drawing were done using GraphPad 6.0 and R version 3.6.2 (http://www.R‐project.org/). Statistical significance was set at *p* < 0.05. All methods complied with pertinent guidelines and regulations.

## 3 RESULTS

### 3.1 Patient characteristics

The training cohort included 35,241 patients who were diagnosed with EEC and adenocarcinoma between 2004 and 2015. For the validation cohort curated from the First Affiliated Hospital of Anhui Medical University, there were a total of 450 suitable patients from 2008 to 2023, 376 of whom underwent active follow‐up, for a 16.44% attrition rate. A preponderance of patients within the training cohort were aged between 50 and 70 years (65.60%). The age distribution followed a similar pattern in the validation cohort. Most of the patients in both cohorts were married, accounting for 56.60% and 100.00%, respectively (due to the small number of widowed patients in the follow‐up cohort, they were not included in the analysis). Regarding tumor characteristics, both cohorts had a primary diagnosis that was most often stage I, low‐to‐middle grade, with a tumor size <4. The OS rates were 11.50% and 10.90% in the training and validation cohorts, respectively. Comprehensive details are provided in Table 1.

**TABLE 1:** Patient characteristics in the training cohort and validation cohort.

	Training cohort (*N* = 35,241)		Validation cohort (*N* = 376)	
	*N*	%	*N*	%
Age				
X < 50	4810	13.60	78	20.70
50 ≤ X < 70	23,112	65.60	283	75.30
70 ≤ X	7319	20.80	15	4.00
Race				
White	29,528	83.8	0	0.00
Black	2276	6.50	0	0.00
Others	3437	9.80	376	100.00
Marriage				
Married	19,933	56.60	376	100.00
Single	6711	19.00	0	0.00
Divorced/separated	4174	11.80	0	0.00
Widowed	4423	12.60	0	0.00
Grade				
Low grade	19,632	55.70	151	40.20
Middle grade	11,845	33.60	184	48.90
High grade	3764	10.70	41	10.90
Tumor size				
X < 4	22,534	63.90	264	70.20
X ≥ 4	12,707	36.10	112	29.80
Stage				
I	32,095	91.10	336	89.40
II	3146	8.90	40	10.60
Radiotherapy				
No	27,815	78.90	272	72.30
Yes	7426	21.10	104	27.20
Chemotherapy				
No	34,242	97.20	257	68.40
Yes	999	2.80	119	31.60
Survival state				
Survive	31,180	88.50	335	89.10
Death	4061	11.50	41	10.90

### 3.2 Nomogram

To identify prognostic factors associated with overall survival (OS) in endometrioid endometrial carcinoma (EEC) patients and adenocarcinoma patients, univariate and multivariate Cox proportional hazards regression analyses were conducted. The results of the univariate and multivariate Cox regression analyses are summarized in Table 2. Several variables, including age, race, marital status, tumor grade, tumor stage, tumor size, and chemotherapy, were independent prognostic factors for OS.

**TABLE 2:** Univariate and multivariate analysis of OS in the training cohort.

Characteristics	Univariate		Multivariate	
	Hazard ratios (95% CI)	*p*	Hazard ratios (95% CI)	*p*
Age				
X < 50	Reference		Reference	
50 ≤ X < 70	2.001 (1.732–2.311)	<0.001	1.9944 (1.680–2.249)	<0.001
70 ≤ X	7.670 (6.643–8.856)	<0.001	6.254 (5.384–7.265)	<0.001
Race				
White	Reference		Reference	
Black	1.600 (1.441–1.777)	<0.001	1.361 (1.224–1.514)	<0.001
Others	0.659 (0.581–0.748)	<0.001	0.831 (0.732–0.944)	0.004
Marriage				
Married	Reference		Reference	
Single	1.263 (1.156–1.380)	<0.001	1.402 (1.281–1.534)	<0.001
Divorced/Separated	1.500 (1.360–1.655)	<0.001	1.445 (1.309–1.595)	<0.001
Widowed	3.210 (2.978–3.460)	<0.001	1.710 (1.578–1.852)	<0.001
Grade				
Low grade	Reference		Reference	
Middle grade	1.731 (1.614–1.856)	<0.001	1.389 (1.293–1.492)	<0.001
High grade	3.194 (2.942–3.467)	<0.001	2.214 (2.026–2.420)	<0.001
Tumor size(cm)				
X < 4	Reference		Reference	
X ≥ 4	1.800 (1.692–1.914)	<0.001	1.549 (1.454–1.651)	<0.001
Stage				
I	Reference		Reference	
II	2.011 (1.847–2.190)	<0.001	1.557 (1.423–1.705)	<0.001
Radiotherapy				
No	Reference		Reference	
Yes	1.627 (1.521–1.741)	<0.001	0.974 (0.904–1.050)	0.493
Chemotherapy				
No	Reference		Reference	
Yes	2.037 (1.757–2.362)	<0.001	1.313 (1.126–1.530)	<0.001

**FIGURE 2:**
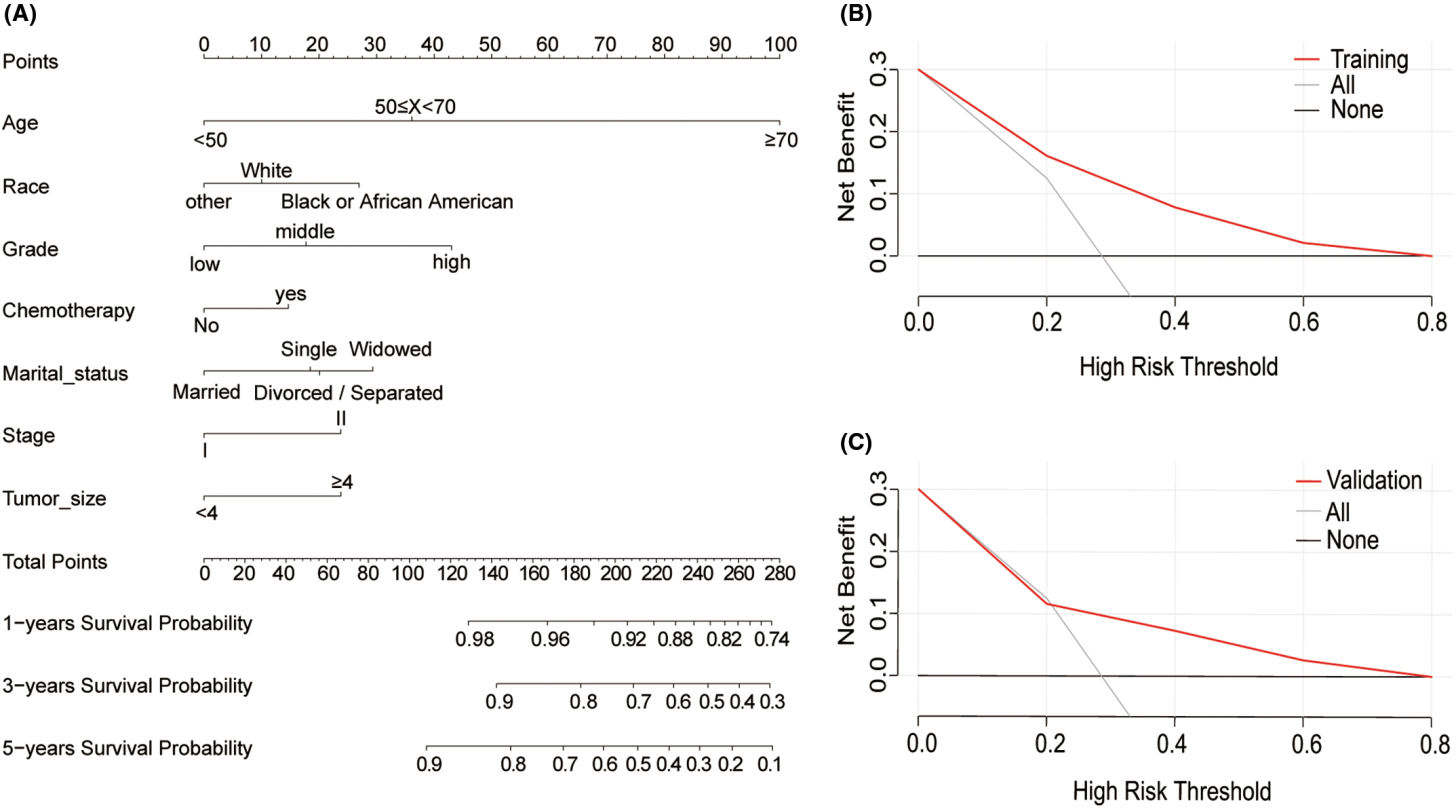
Establishment and validation of the nomogram. (A) Nomogram predicting the 1‐, 3‐, and 5‐year OS in patients with EEC and adenocarcinoma; DCA of the nomogram in (B) the training cohort and (C) the validation cohort.

**FIGURE 3:**
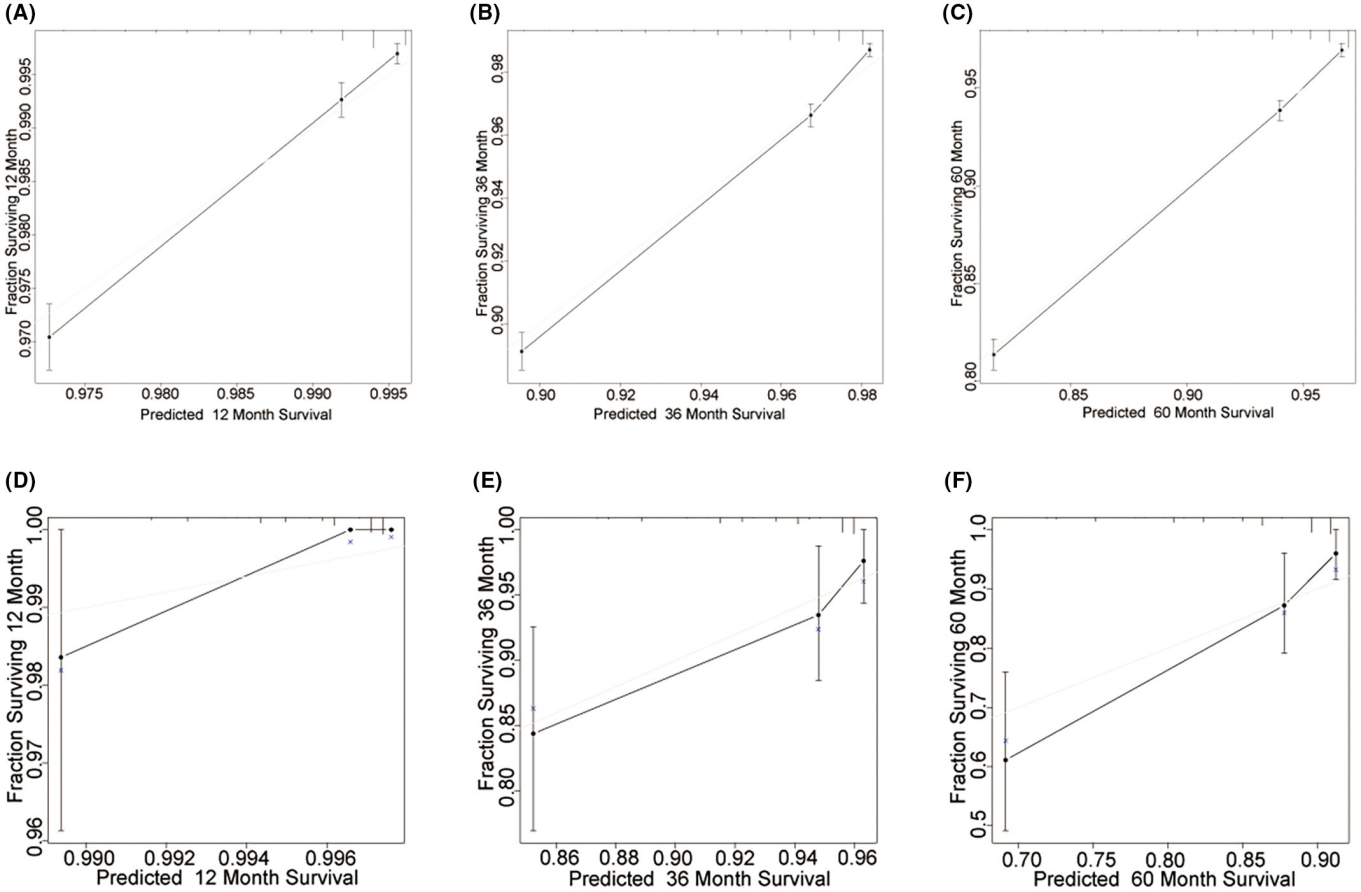
Calibration plots for (A) the 1‐year OS nomogram in the training cohort; (B) the 3‐year OS nomogram in the training cohort; (C) the 5‐year OS nomogram in the training cohort; (D) the 1‐year OS nomogram in the validation cohort; (E) the 3‐year OS nomogram in the validation cohort; and (F) the 5‐year OS nomogram in the validation cohort.

**FIGURE 4:**
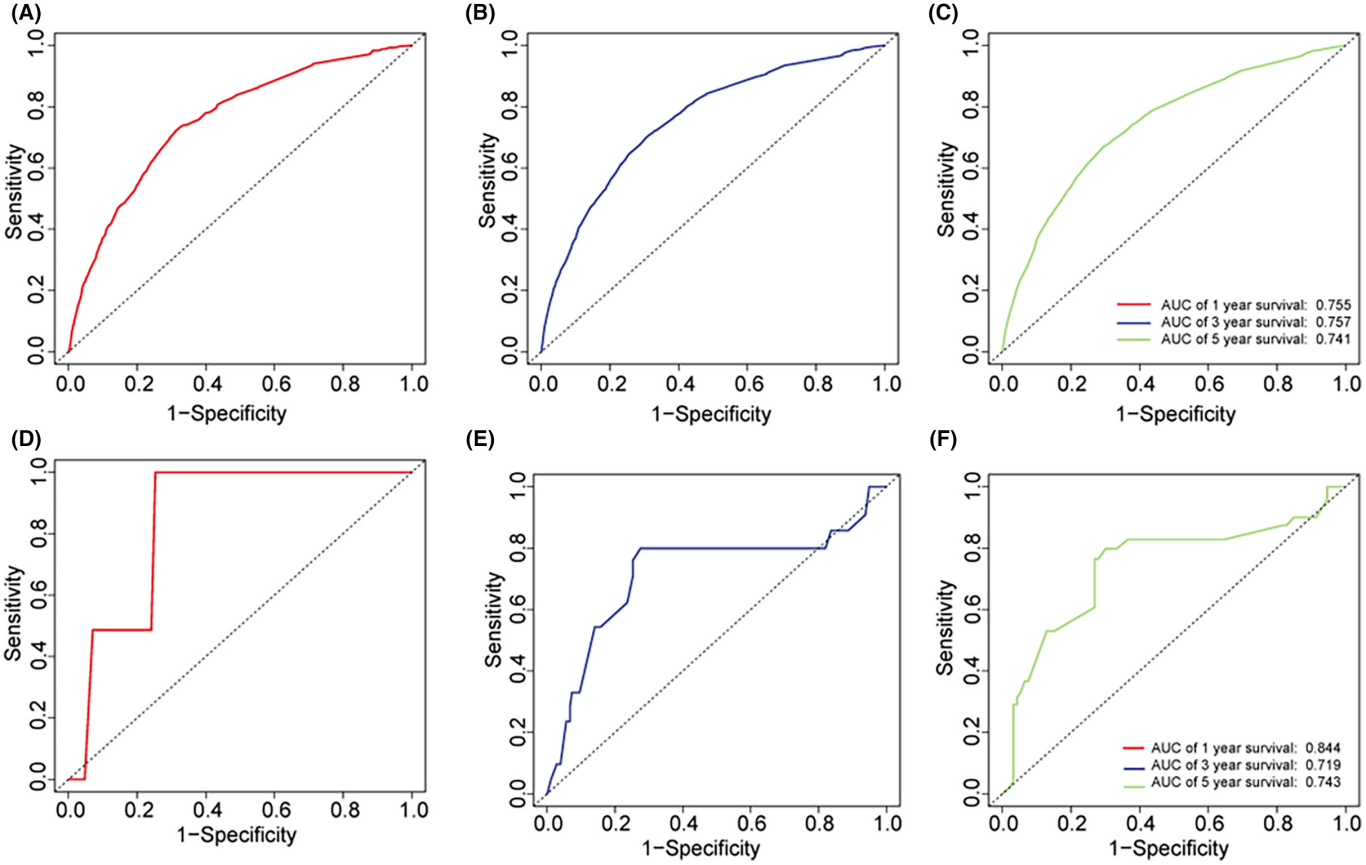
ROC curves for (A) the 1‐year OS nomogram in the training cohort; (B) the 3‐year OS nomogram in the training cohort; (C) the 5‐year OS nomogram in the training cohort; (D) the 1‐year OS nomogram in the validation cohort; (E) the 3‐year OS nomogram in the validation cohort; and (F) the 5‐year OS nomogram in the validation cohort.

The independent prognostic indicators for OS in patients with EEC and adenocarcinoma from the Cox proportional hazard regression analysis were utilized to construct a nomogram, as depicted in Figure 2A. In the random samples from both the SEER and external datasets, the nomogram's C‐index was recorded as 0.743 and 0.720, respectively. The DCA for the nomogram substantiated its clinical utility by showing that the model offered a higher net benefit over a broad spectrum of probability thresholds than the other two scenarios (Figure 2B,C). Calibration plots demonstrated good concordance between the nomogram‐predicted 1‐, 3‐, and 5‐year OS and the actual outcomes in both the training and validation cohorts (Figure 3). The ROC curves for the prediction model are shown in Figure 4. The area under the ROC curve (AUC) values for 1‐, 3‐, and 5‐year OS was 0.755, 0.757, and 0.741, respectively, for the SEER dataset and 0.844, 0.719, and 0.743 for the external dataset. These data illustrate that the model had robust discriminative capabilities.

### 3.3 Analysis of the correlation between tumor grade and OS

**FIGURE 5:**
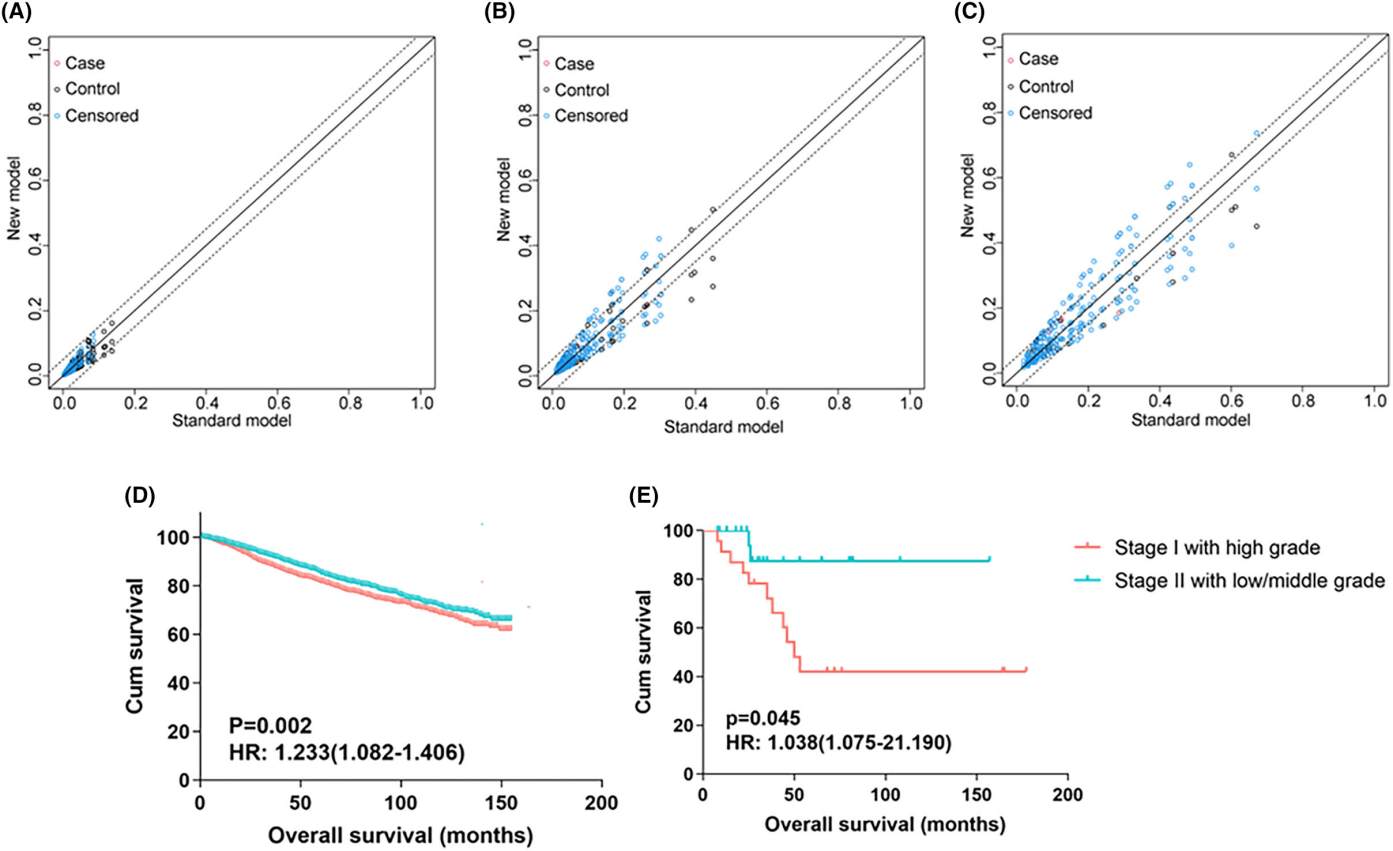
NRI for (A) the 1‐year OS nomogram in the training cohort; (B) the 3‐year OS nomogram in the training cohort; (C) the 5‐year OS nomogram in the training cohort; and Kaplan–Meier curves between tumor grade and OS after PSM in (D) the training cohort and (E) the validation cohort.

Tumor grade emerged as the most critical histopathological factor in the nomogram. This is a novel histopathological factor integrated into the FIGO staging. Therefore, we conducted a more in‐depth investigation into grade. First we assessed the influence of tumor grade on the nomogram model. The 3‐year and 5‐year NRI values for the new model that included tumor grade and the model without tumor grade in the SEER dataset came in at 0.10 and 0.09, respectively (Figure 5A–C). These values indicate a significant enhancement in the model by adding tumor grade. Next, to substantiate the significant impact of tumor grade on the prognosis of endometrial cancer patients, we carried out PSM on the training and validation sets. After PSM, no significant disparities were observed in the confounding factors between stage I high‐grade patients and stage II low/middle‐grade patients (Tables 3 and 4). Lastly, the survival analysis revealed a markedly worse prognosis for patients with high‐grade stage I disease (Figure 5D,E).

**TABLE 3:** Baseline characteristics after the PSM of training cohort.

	Stage II with low/ middle grade		Stage I with high grade		*p*
	*N*	%	*N*	%	
Age					
X < 50	243	10.4	240	10.3	0.504
50 ≤ X < 70	1533	65.9	1568	67.4	
70 ≤ X	551	23.7	519	22.3	
Race					
White	1926	82.80	1932	83.00	0.600
Black	183	7.90	194	8.30	
Others	218	9.40	201	8.60	
Marriage					
Married	1263	54.30	1252	53.80	0.964
Single	442	19.00	445	19.10	
Divorced/Separated	290	12.50	301	12.90	
Widowed	332	14.30	329	14.10	
Tumor size					
X < 4	1055	45.30	1064	45.70	0.791
X ≥ 4	1272	54.70	1263	54.30	
Radiotherapy					
No	1095	47.10	1072	46.10	0.499
Yes	1232	52.90	1255	53.90	
Chemotherapy					
No	2182	93.80	2160	92.80	0.197
Yes	145	6.20	167	7.20	

**TABLE 4:** Baseline characteristics after the PSM of validation cohort.

	Stage II with low/middle grade		Stage I with high grade		*p*
	*N*	%	*N*	%	
Age					
X < 50	4	17.40	4	17.40	1.000
50 ≤ X < 70	19	82.60	19	82.60	
70 ≤ X	0	0.00	0	0.00	
Marriage					
Married	23	100.00	23	100.00	1.000
Single	0	0.00	0	0.00	
Divorced/Separated	0	0.00	0	0.00	
Widowed	0	0.00	0	0.00	
Tumor size					
X < 4	10	43.50	10	43.50	1.000
X ≥ 4	13	56.50	13	56.50	
Radiotherapy					
No	11	47.80	11	47.80	1.000
Yes	12	52.20	12	52.20	
Chemotherapy					
No	7	30.40	7	30.40	1.000
Yes	16	69.60	16	69.60	

## 4 DISCUSSION

Endometrial cancer is the second most common gynecological malignancy worldwide. Current projections are that endometrial cancer may surpass colorectal cancer to become the third most prevalent cancer and fourth leading cause of cancer mortality in women by 2030.^15^ The primary treatment for endometrial cancer is total hysterectomy with bilateral salpingo‐oophorectomy. In premenopausal women with early stage endometrial cancer, ovarian preservation may be considered to avoid the consequences of surgical menopause, without compromising survival.^12^, ^13^ Hence, help optimize the treatment for the majority of endometrial cancer patients, we performed a prognostic analysis and constructed a nomogram for the principal histological subtypes of EEC and adenocarcinoma after total hysterectomy.^16^

We analyzed eight variables from the SEER database to evaluate factors impacting the prognosis of patients with EEC and adenocarcinoma. A nomogram for predicting OS was then developed based on multivariate Cox proportional hazards regression. The OS nomogram incorporated age, race, marital status, tumor size, stage, tumor grade, and chemotherapy as prognostic variables. Our nomogram is highly innovative and practical for two reasons. First, unlike the FIGO staging, our nomogram integrates demographic factors, tumor characteristics, and treatment as independent prognostic factors for OS. These variables can be easily obtained by clinicians. When predicting the prognosis of patients with EEC and adenocarcinoma, our nomogram can reduce the biases inherent to patient demographics and variable treatment methodologies. Second, our nomogram was validated in an external dataset. External validation gauges the nomogram's predictive capability in various populations, establishing its applicability to different groups.^17^

The nomogram C‐index for both the SEER database and the external dataset, obtained through random sampling, was greater than 0.70, indicating that our nomogram has good discriminative ability.^18^, ^19^ The calibration plots showed good agreement between the predicted and observed OS probabilities, with ideal alignment along the 45‐degree line, indicating that the nomogram was well‐calibrated.^20^ Therefore, our nomogram is accurately calibrated to forecast 1‐year, 3‐year, and 5‐year OS. DCA was performed to evaluate the clinical utility of the nomogram by quantifying the net benefit across a range of threshold probabilities.^21^ DCA showed that the nomogram provides improved clinical utility and net benefit over existing methods for predicting survival in EEC and adenocarcinoma patients.

Age is an acknowledged high‐risk factor for endometrial cancer.^22^ According to our nomogram, age bears the most weight, and previous research indicates that in postmenopausal women, progesterone deficiency leading to unopposed estrogen excess significantly elevates to endometrial cancer risk due to faster endometrial proliferation.^3^ Further, older patients often have worse physical fitness, more comorbidities, and lower tolerance to supplementary therapy than younger ones, resulting in a worse prognosis.^23^, ^24^

Statistics indicate that 7% of patients diagnosed with endometrial cancer are younger than 45, and a substantial proportion of these patients have reproductive demands.^25^ For young endometrial cancer patients with reproductive demands, fertility‐preserving treatment has emerged as a viable option. Before opting for fertility‐preserving treatment, risk factors for endometrial cancer, such as lymph node metastasis, obesity, and polycystic ovary syndrome, should be evaluated.^26^ Recent studies have provided further evidence supporting the choice of fertility‐preserving treatment for endometrial cancer patients. For instance, Ida Pino et al. developed a nomogram with relatively accurate predictive capability for high‐risk factors associated with lymph node involvement in endometrial cancer.^27^ Additionally, molecular classification might become the standard approach to endometrial cancer management.^28^ Giorgio Bogani et al. combined radiomic features with molecular features to enhance the decision‐making process in patients with endometrial cancer.^29^

For young, low‐risk endometrial cancer patients, conservative treatments such as hormone therapy under close surveillance may be considered at first, with surgical intervention pursued after the woman passes childbearing age.^30^ Accumulating evidence suggests that employing conservative approaches and close monitoring until delivery does not compromise the survival outcomes of endometrial cancer patients. For patients without male partners or those unwilling to cryopreserve embryos, oocyte preservation can be achieved through vitrification and slow cooling techniques.^31^, ^32^ Notably, vitrification techniques mitigate the risk of multiple pregnancies and ovarian hyperstimulation syndrome.^31^, ^32^ For babies conceived by assisted reproductive technologies, including oocyte cryopreservation, the long‐term health implications warrant further investigation.

Marital status, another demographic characteristic, also significantly impacts endometrial cancer prognosis. Research on the influence of marital status on cancer has revealed that married patients tend to be diagnosed earlier.^33^ Furthermore, the psychological wellness and social support of married patients can exert antitumor effects by regulating the tumor immune microenvironment.^34^

Interestingly, among histopathological factors, tumor grade has the strongest effect on prognosis, exceeding even that of tumor stage. Endometrioid carcinoma can be classified into three grades of structural complexity.^35^ Extensive evidence indicates that patients with high‐grade tumors have a worse prognosis than patients with low/middle‐grade tumors.^36^, ^37^ Compared to the 2009 version of the FIGO staging system, which does not consider grade, the new FIGO staging system, classifies high‐grade EEC directly into stage II due to its invasive nature.^4^, ^7^ In our study, further PSM analysis showed that the prognosis of patients with stage I high‐grade disease was significantly worse than that of patients with stage II low/middle‐grade disease. This finding is consistent with the new FIGO staging system, confirming the accuracy of our model in risk prediction and the rationality of the new FIGO staging system. Tumor size made quite a large contribution to the nomogram output, similar to that of stage. Tumor size has a significant impact on the prognosis of patients with endometrial cancer,^38^, ^39^ another finding was validated by our nomogram model. The tumor size can be measured more accurately through preoperative imaging, which is clinically relevant for the selection of surgical procedures and adjuvant treatments for endometrial cancer.^40^ In the future, with more data validation, tumor size could serve as a clinical indicator for substaging within the FIGO staging system to further guide clinical treatment.

Although we constructed a meaningful nomogram and validated the new FIGO staging system using both training and validation datasets, our study has several limitations. First, as a retrospective study, it gathered data from datasets and patients with missing data for the variables of interest were excluded, which could introduce selection bias. Second, we lacked key indicators, especially details about radiotherapy doses and chemotherapy regimens. For example, in the SEER database, radiotherapy is only categorized as “Yes” or “No,” which might diminish the impact of radiotherapy on survival. Third, prognostic factors such as LVSI are missing.

## 5 CONCLUSIONS

In summary, age, race, marital status, tumor grade, tumor size, and chemotherapy all serve as independent prognostic factors for OS in patients with EEC and adenocarcinoma. Our OS nomogram that was built from multivariate Cox proportional hazard regression analysis of a training set and was validated in an external dataset exhibits strong predictive power and clinical utility. Furthermore, this work can serve as the foundation for validating the 2023 FIGO staging system.

## AUTHOR CONTRIBUTIONS

**Yifan Feng:** Data curation (lead); software (lead); writing – original draft (lead). **Fulu Miao:** Data curation (lead); formal analysis (lead). **Yuyang Li:** Data curation (equal); formal analysis (equal). **Min Li:** Project administration (equal); validation (equal). **Yunxia Cao:** Project administration (lead); validation (lead).

## FUNDING INFORMATION

The Clinical Medical Research Transformation Project of Anhui Province (202204295107020029).

## CONFLICT OF INTEREST STATEMENT

The authors declare that no competing interests exists.

## ETHICS STATEMENT

This study received ethical approval from the First Affiliated Hospital of Anhui Medical University Ethics Committee and the informed consent was waived.

## Data Availability

All data are available from the corresponding authors upon reasonable request.
